# Clinical outcomes of COVID-19 in patients undergoing chronic hemodialysis and peritoneal dialysis

**DOI:** 10.1590/2175-8239-JBN-2021-0261en

**Published:** 2022-05-25

**Authors:** Fernanda Salomão Gorayeb-Polacchini, Heloisa Cristina Caldas, Mario Abbud-Filho

**Affiliations:** 1 Hospital de Base de São José do Rio Preto, Faculdade de Medicina de São José do Rio Preto, Divisão de Nefrologia, São José do Rio Preto, SP, Brasil.; 2 Faculdade de Medicina de São José do Rio Preto, Laboratório de Imunologia e Transplante Experimental, São José do Rio Preto, SP, Brasil.

**Keywords:** SARS-CoV-2, COVID-19, Dialysis, SARS-CoV-2, COVID-19, Diálise

## Abstract

**Background:**

The reported incidence and fatality rate of the severe acute respiratory syndrome coronavirus 2 in patients receiving chronic dialysis are higher than in the general population. We sought to study the outcomes following coronavirus disease 2019 (COVID-19) diagnosis in patients undergoing chronic hemodialysis (HD) or peritoneal dialysis (PD) in a single center in Brazil.

**Methods:**

Of the 522 patients on dialysis evaluated between March 1, 2020, and October 1, 2021, those presenting symptoms or with a history of close contact with COVID-19 patients were tested with reverse-transcription polymerase chain reaction of samples from nasopharyngeal swabs.

**Results:**

Of the 522 patients, 120 were positive for COVID-19 infection, of which 86% were on HD and 14% in the PD program. The incidence per 10,000 inhabitants was higher in the HD group than in the PD group (2,423.5 vs. 1,752.5). The mortality per 10,000 inhabitants (470.5 vs. 927.8) and the fatality rate (19.4 vs. 52.9%, p = 0.005) were higher in the PD group. The PD group also had a higher need for hospitalization, intensive care, and mechanical ventilation.

**Conclusions:**

We advise caution when considering strategies to transfer patients from HD to the PD program to minimize the risk of COVID-19 for patients on HD.

## Resumo

**Antecedentes:**

A incidência e a taxa de letalidade da síndrome respiratória aguda grave por coronavírus 2 relatadas em pacientes em diálise crônica são mais elevadas do que na população em geral. Procuramos estudar os desfechos após o diagnóstico da doença por coronavírus 2019 (COVID-19) em pacientes submetidos à hemodiálise crônica (HD) ou diálise peritoneal (DP) em um único centro no Brasil.

**Métodos:**

Dos 522 pacientes em diálise avaliados entre 1º de Março de 2020 e 1º de Outubro de 2021, aqueles que apresentaram sintomas ou tiveram histórico de contato próximo com pacientes com COVID-19 foram testados com reação em cadeia da polimerase de transcrição reversa por meio de amostras de esfregaços nasofaríngeos.

**Resultados:**

Dos 522 pacientes, 120 foram positivos para infecção por COVID-19, dos quais 86% estavam em HD e 14% no programa de DP. A incidência por 10.000 habitantes foi maior no grupo HD do que no grupo DP (2.423,5 vs. 1.752,5). A mortalidade por 10.000 habitantes (470,5 vs. 927,8) e a taxa de letalidade (19,4 vs. 52,9%, p = 0,005) foram mais elevadas no grupo DP. O grupo DP também apresentou uma maior necessidade de hospitalização, terapia intensiva e ventilação mecânica.

**Conclusões:**

Recomendamos cautela ao considerar estratégias de transferência de pacientes do programa de HD para o de DP a fim de minimizar o risco de COVID-19 para pacientes em HD.

## Introduction

The coronavirus 2019 (COVID-19) outbreak had considerable effects on the healthcare system and the global economy. Accumulating evidence shows that patients on chronic dialysis are among the most vulnerable to COVID-19^1,2^, with multiple studies reporting fatality rates above 20%^3,4,5^. Although infection rates among dialysis patients tend to follow local patterns, the incidence of COVID-19 is higher in this population than in the general population, probably due to increased testing, symptoms screening, need for medical care, and sharing of public transportation for routine travel to the dialysis facility^6-8^.

In most countries, dialysis is mostly delivered as in-center hemodialysis (HD), but the minority of home dialysis patients, either HD or peritoneal dialysis (PD), have comparable outcomes^2,9^.

COVID-19 is especially a problem for patients undergoing in-center HD. Frequent trips to the dialysis facility and grouping patients with advanced age and comorbidities, promote a high-risk situation for COVID-19 transmission and related morbidity and mortality^7,8,10^.

Compared with HD, PD can be performed at home and staff can conduct telemedicine consultations and prescriptions, which may reduce the risk of COVID-19 infection.^11^ Because the risk of COVID-19 is lower with home dialysis than with in-center dialysis, home dialysis protects patients from COVID-19-related morbidity and mortality^2,12^.

Patients with severe COVID-19 may develop respiratory failure, and current COVID-19 guidelines recommend conservative fluid management, because hypervolemia may worsen hypoxia.^13^ Compared with intermittent HD, PD may be a better choice for these patients, since it is given more frequently and may result in less hypervolemia.

However, PD may have some disadvantages during a COVID-19 infection. The peritoneal fluid increases intraperitoneal pressure, which may compromise pulmonary function and peritonitis can result in decreased ultrafiltration rate and hypervolemia, thus worsening hypoxia^14^. Despite reports that severe acute respiratory syndrome coronavirus 2 (SARS-CoV-2) can reach the dialysis effluent in patients with PD^15^ and that antibodies were found in PD dialysate, it is still unknown whether SARS-CoV-2 can break the gastrointestinal mucosal barrier and increase the risk for peritonitis^16^.

In February 2020, the first COVID-19 case was detected in Brazil in the city of São Paulo. The first case in São José do Rio Preto, in the state of São Paulo, was detected in March 2020. With more than 145,000 patients on chronic dialysis programs in Brazil and only 7.3% of them on PD, strategies need to be developed to mitigating the effect of COVID-19 in this population^6^.

Studies describing COVID-19 outcomes in patients with PD compared with those with in-center HD are lacking. Nevertheless, these studies are needed because these patients have similar risk factors for poor COVID-19 outcomes but different risk factors for infection.

## Materials and Methods

We prospectively analyzed the outcomes of patients with COVID-19 undergoing HD (all in-center patients) and PD in a dialysis unit that is a reference center for more than two million inhabitants, from March 1, 2020, to October 1, 2021. All patients with COVID-19 were confirmed by SARS-CoV-2 reverse-transcription polymerase chain reaction (RT-PCR) test using from nasopharyngeal swab samples.

Of the total 522 patients (425 in the HD group and 97 in the PD group), patients who had symptoms of the disease or reported close contact with COVID-19 were tested. Data were obtained from the online Hospital Registration System and we analyzed the demographic characteristics, clinical outcomes, laboratory tests, and incidence, mortality, and fatality rates of the HD and DP patients who tested positive for COVID-19.

The Ethics Committee approved the applied protocol (No. 4212395).

### Statistical Analysis

Quantitative variables (such as age and treatment duration) are presented as means ± standard deviation (SD), while categorical variables (such as sex and treatment modality) are presented as frequencies (percentage). For between group comparison, the χ2 test was used for categorical variables and Student’s t-test (data with normal distribution) or the Mann-Whitney test (data with non-normal distribution) was used for continuous variables. Data analyses were performed using StatsDirect 3.0 software. P < 0.05 values are considered significant.

## Results

Of the 522 patients on maintenance dialysis, 120 (23%) were diagnosed with COVID-19 during the 20-month period of the pandemic, of which 103/425 were on HD and 17/97 were on PD.

As shown in Table 1, the mean age of the total sample was 60.3 years, 56.6% were male, and 81.6% were white. Epidemiological analysis of positive COVID-19 patients revealed that 49% had contact with the disease at home, 26% were unaware of any close contact, 16% had contact during public transport to the dialysis unit, and 9% had contact during hospitalization.

**Table 1:** Demographic characteristic and outcomes of patients with COVID-19

Characteristics	Total (n = 120)	HD (n =103)	PD (n = 17)	P value
Age (years),	60.3 ± 14.6	59.5 ± 15	65 ± 13	0.16
Sex, M [n (%)]	68 (56.6)	57 (55.3)	11 (64.7)	0.6
Race (W/NW)	98/22	85/18	13/4	0.51
**Coexisting disorder, [n (%)]**				
Cardiovascular disease	48 (40)	41 (39.8)	07 (41.17)	1
Hypertension	112 (93.3)	98 (95.1)	14 (82.3)	0.08
Diabetes mellitus	57 (47.5)	51 (49.5)	06 (35.2)	0.3
Lung disease	33 (27.5)	29 (28.1)	04 (23.5)	0.7
Cancer	15 (12.5)	12 (11.6)	03 (17.6)	0.4
**Dialysis (months)**	44.2 ± 41.5	48.4 ± 43	18.6 ± 13.4	**0.005**
**Treatments, [n (%)]**				
Oxygen therapy	55 (45.8)	42 (40.7)	13 (76.4)	**0.008**
Mechanical ventilation	24 (20)	16 (15.5)	08 (47)	**0.006**
Hospitalization, [n (%)]	60 (50)	47 (45.6)	13 (76.4)	**0.03**
Hospitalization period (days)	6 ± 8.2	5.3 ± 8.4	9.5 ± 6.7	**0.01**
ICU, [n (%)]	29 (24.1)	21 (20.3)	08 (47)	**0.02**
Thrombosis [n (%)]	16 (13.3)	15 (14.5)	1 (5.8)	0.4
*Incidence rate/10,000	2,298.8	2,423.5	1,752.5	-
*Mortality rate/10,000	555.5	470.5	927.8	-
*Fatality rate, %	29 (24.1)	20 (19.4)	09 (52.9)	**0.005**
**ǂ** Mild disease [n (%)]	57 (47.5)	49 (47.5)	4 (23.5)	0.1
**ǂ** Moderate disease [n (%)]	27 (22.5)	23 (22.3)	4 (23.5)	1
**ǂ** Severe disease [n (%)]	36 (30)	27 (26.2)	9 (52.9)	**0.03**
Death [n (%)]	29 (24.1)	20 (19.4)	09 (52.9)	**0.005**
Death [n (%)] in 3 months post COVID-19	4 (3.3)	4 (3.8)	0 (0)	1
Death [n (%)] after 3 months post COVID-19	5 (4.1)	5 (4.8)	0 (0)	1

* Calculations: The incidence, mortality, and case fatality rates were calculated as follows: Incidence = number of cases from 03/01/2020 until the end of the current report 10/01/2021 / number of exposed people per 10,000. Mortality = number of deaths due to COVID-19/number of exposed people per 10,000. Fatality = (number of confirmed deaths due to COVID-19/number of confirmed COVID-19 cases)

* 100. HD: Hemodialysis; PD: Peritoneal dialysis; ǂ Mild disease: patients not requiring hospitalization; Moderate disease: patients requiring hospitalization or oxygen necessity; Severe disease: intensive care unit (ICU) admission, respiratory failure requiring mechanical ventilation (MV), shock, or death.

No significant difference was found between the two groups concerning age, sex, race, coexisting disorders (Table 1), symptoms (Table 2), or vaccination status during the course of COVID-19 (Table 2). Despite the mean age of patients in the groups being numerically and clinically different (HD 59.5 ±15 vs. PD 65 ± 13, p = 0.16), the difference was not statistically different. The time on dialysis for the HD group was significantly longer than for the PD group (p = 0.005).

**Table 2:** Symptoms, examinations, and vaccination characteristics of patients with COVID-19

	Total (n = 120 )	HD (n = 103)	PD (n = 17)	P value
**Symptoms, [n (%)]**				
Fever	73 (60.8)	65 (63.1)	08 (47)	0.2
Cough	70 (58.3)	58 (56.3)	12 (70.5)	0.3
Dyspnea	57 (47.5)	49 (47.5)	08 (47.0)	1
Diarrhea	28 (23.3)	25 (24.2)	03 (17.6)	0.3
Asthenia	38 (31.6)	29 (28.1)	09 (52.9)	0.08
**Laboratory findings**				
Hb (g/dL)	10.8 ± 2	11 ± 2	9.6 ± 1.7	**0.01**
Platelet - per mm^3^	153,150.3 ± 126,000	151,193± 121,000	170,000± 164,000	0.6
Leukocytes - per mm^3^	5,358 ± 2,564	5,079.6 ± 2,070	7,724± 4,624	**0.0006**
Lymphocytes - per mm^3^	975.2 ± 530	1009 ± 532	684.5 ± 426	**0.04**
Neutrophils - per mm3	3,742.5 ± 2,286	3,488.5 ± 1,860	5,901 ± 4,028	**0.0004**
N:L	5.65 ± 8.6	4.5 ± 4	15.3 ± 23	**0.001**
CRP, mg/dL	8.13 ± 9.6	7.4 ± 9.2	14 ± 11	**0.02**
AST, u/L	27 ± 26	25 ± 25	43 ± 31	**0.02**
ALT, u/L	20.5 ± 24.6	19.6 ± 25.3	27.4 ± 18	**0.03**
Total serum bilirubin, mg/dL	0.3 ± 0.2	0.29 ± 0.1	0.4 ± 0.3	**0.04**
Gamma GT, ui/L	107.6 ± 219	112 ± 223	70 ± 68	0.83
ALP, ui/L	133 ± 103	137.5 ± 106.5	91.4 ± 42	0.1
D-dimer, ug/mL	2 ± 2.3	1.97 ± 2.3	2.52 ± 1.8	0.1
Lactic dehydrogenase, u/L	289 ± 133.5	273 ± 101	424 ± 127	**0.0001**
Albumin, g/dL	3.7 ± 0.6	3.76 ± 0.5	3.55 ± 0.7	0.2
**Vaccination, n**				
Coronavac (SINOVAC) 1º dose or incomplete 2º dose (complete)				
	08 (6.66)	06 (5.8)	02 (11.7)	0.7
	11 (9.1)	09 (8.7)	02 (11.7)	0.7
AstraZeneca AZD1222 (ChAdOx1)				
1º dose or incomplete	35 (29.1)	29 (28.1)	06 (35.2)	0.5
2º dose (complete)	0 (0)	0 (0)	0 (0)	
Not vaccinated	66 (55)	59 (57.2)	7 (41.1)	0.3

Data are reported as mean ± SD for quantitative variables and n (%) for nominal parameters. HD: Hemodialysis. PD: Peritoneal dialysis. CRP: C-reactive protein. Hb: hemoglobin. LDH: lactate dehydrogenase. N:L: neutrophils to lymphocytes ratio. AST: aspartate aminotransferases. ALT: alanine aminotransferases. ALP: Alkaline phosphatase.

Mean hemoglobin and lymphocyte values were lower in the PD group. Leukocyte count, neutrophil count, neutrophil lymphocyte ratio (N:L), and levels of C-reactive protein (CRP), lactic dehydrogenase, aspartate aminotransferase, alanine aminotransferase, and bilirubin were higher in the PD group than in the HD group (Table 2).

The clinical outcomes, development of severe disease, need for oxygen therapy, mechanical ventilation, hospitalization, hospitalization period, need for intensive care, and death were higher in the PD than in the HD group (Table 1).

The incidence rate (per 10,000) was higher in the HD group (2,423.5 vs. 1,752.5), while the mortality rate per 10,000 (470.5 vs. 927.8) and fatality rate were higher in the PD group (19.4% vs. 52.9%; p = 0.005).

## Discussion

While there are several studies reporting the impact of COVID-19 infection in chronic HD patients,^7,17-19^ information on PD patients is still lacking.^7^ Therefore, in the present study, we evaluated the impact of the disease on both groups of dialysis patients.

Our results showed that the incidence, mortality, and fatality rates in both HD and PD groups by far exceed those observed in the general Brazilian population (incidence of 1,026.7/10,000 inhabitants, mortality of 28.6/10,000 inhabitants, and lethality rate of 2.8%)^20^, confirming the high risk of poor outcomes in the population on maintenance dialysis.

Although the PD group stays at home and can maintain social distancing, health care utilization is higher than average in this population than the general population, due to comorbidities and end-stage renal disease, which can explain the higher incidence in the PD group compared with the general population.^2^

As expected, in agreement with other studies, the incidence of COVID-19 in our study was lower in the PD than in the HD group.^21-23^ The HD group was unable to adhere to isolation recommendations, and 16% reported having contact with COVID-19 in public transportation on the way to the dialysis facility. On the contrary, PD patients, received telemedicine consultation during the COVID-19 pandemic.^24^ Moreover, telehealth has played a pivotal role in the current pandemic and should be implemented whenever possible; however, the quality of evidence is controversial.^25-26^

Studies evaluating the impact of COVID-19 on PD and HD patients have conflicting results^2,8,22,23,27^. Our study found higher hospitalization, oxygen therapy need, mechanical ventilation, mortality, and fatality rates (52.9% vs. 19.4%; p = 0.005) in the PD group than in the HD group. Other studies also found a higher COVID-19 fatality rate in the PD group than in the HD group (33% vs. 29.6%^22^ and 45.6% vs. 34.5%^23^), corroborating our previous study that showed that the PD group had a higher rate of severe COVID-19 than the HD group.^8^ In contrast, Weinhandl et al reported that the PD group had a much lower risk of hospitalization than the HD group^27^. Hsu et al did not find significant differences in the morbidity and mortality rates between the HD and PD groups with COVID-19^2^.

Our study found significantly worse laboratory test results in the PD group, suggesting a higher disease severity in this group, in accordance to other reports.^8,28,29^ A study on dialysis and COVID-19 demonstrated a correlation between higher initial values of white blood cells, LDH, and CRP and several inflammatory markers and disease severity and death.^28^ Broseta et al. also showed that higher LDH and CRP levels at admission were associated with higher COVID-19 mortality risk in the dialysis population.^29^

Limitations of this study include the relatively small sample size and the observational nature of the study, the low number of COVID-19 tests in PD patients, and the possibility of asymptomatic patients not being tested.

## Conclusion

In conclusion, the incidence, mortality, and fatality rates of COVID-19 in HD and PD patients were substantially higher than in the general population, and the PD group had worse outcomes than the HD group. We advise caution when considering strategies to transfer patients from HD to the PD program to minimize the risk of COVID-19 in patients on HD.
